# Not “Pulling up the Ladder”: Women Who Organize Conference Symposia Provide Greater Opportunities for Women to Speak at Conservation Conferences

**DOI:** 10.1371/journal.pone.0160015

**Published:** 2016-07-28

**Authors:** Stephanie Sardelis, Joshua A. Drew

**Affiliations:** Department of Ecology, Evolution and Environmental Biology, Columbia University, 1200 Amsterdam Ave., New York, NY, 10027, United States of America; University of Tuebingen Medical School, GERMANY

## Abstract

The scientific community faces numerous challenges in achieving gender equality among its participants. One method of highlighting the contributions made by female scientists is through their selection as featured speakers in symposia held at the conferences of professional societies. Because they are specially invited, symposia speakers obtain a prestigious platform from which to display their scientific research, which can elevate the recognition of female scientists. We investigated the number of female symposium speakers in two professional societies (the Society of Conservation Biology (SCB) from 1999 to 2015, and the American Society of Ichthyologists and Herpetologists (ASIH) from 2005 to 2015), in relation to the number of female symposium organizers. Overall, we found that 36.4% of symposia organizers and 31.7% of symposia speakers were women at the Society of Conservation Biology conferences, while 19.1% of organizers and 28% of speakers were women at the American Society of Ichthyologists and Herpetologists conferences. For each additional female organizer at the SCB and ASIH conferences, there was an average increase of 95% and 70% female speakers, respectively. As such, we found a significant positive relationship between the number of women organizing a symposium and the number of women speaking in that symposium. We did not, however, find a significant increase in the number of women speakers or organizers per symposium over time at either conference, suggesting a need for revitalized efforts to diversify our scientific societies. To further those ends, we suggest facilitating gender equality in professional societies by removing barriers to participation, including assisting with travel, making conferences child-friendly, and developing thorough, mandatory Codes of Conduct for all conferences.

## Introduction

Representation of women in science, technology, engineering, and math education (STEM) has increased over the past two decades. Approximately 60% of Master’s recipients and between 36% and 48% of PhD recipients are women [1][2][3]). Half of all MD and 52% of all PhD degrees in life sciences are awarded to women [4]. Additionally, women now comprise 50% of the United States workforce [5]. Yet, there is still inequality in women’s representation within STEM fields: women occupy less than 40% of jobs in biology, chemistry, and physics, and only 25% of STEM jobs overall [5]. Women also only hold between 18% and 24% of full-time professor positions [1][2][3]. Further, while women constitute 20% of the earth and environmental sciences field, only 3.8% of publications in *Nature* from that discipline are of women authorship [6]. Such an imbalance is a consequence of the culture of academia, which, regrettably, harbours gender biases against women [7][8][9]).

### The Leaky Pipeline Effect

Gender biases can be subtle or blatant, ranging from social exclusion due to stereotypes to unequal distribution of promotions, awards, and tenure [10]. These biases are self-fulfilling gender schema [11]: underrepresentation of women at senior academic levels may negatively influence the ambitions of junior female scientists [12][13]. While increased exposure to female role models correlates with heightened positive self-conception and reduced stereotype application to young female scientists [14][15], the absence of female scientific role models has been postulated to limit the number of young women entering STEM fields [16].

Without affirmative guidance, a “leaky-pipeline” effect is observed, whereby a gender filter removes women from the academic stream and exclusively allows men to progress [14][16]. This causes the number of women in science to undergo attrition as the academic level increases [17][18]. For example, the leaky pipeline effect has been documented occurring in Sweden, where approximately 45% of PhD candidates were women, but women only held 25% of postdoctoral positions and less than 10% of professor roles [19]. Moreover, men were more likely than women to secure professor roles within the American Institutes for Research upon completing their PhDs [20]. While the leaky pipeline effect is not necessarily intentional or malicious [16], it is likely related to a multitude of factors—such as applying stereotypes to women—cumulating in the aforementioned imbalance. Therefore, higher visibility of female scientists might help to alleviate the leaky pipeline dilemma.

### Gender Perception Gaps

Women in science are confronted by systematic challenges related to gender. Female scientists with identical qualifications and experience are judged to be less competent than male colleagues, are offered less mentoring, and are offered a lower starting salary [21]. Male STEM professors are nearly twice as likely to discuss research when conversing with a male colleague as when talking with a female colleague [22]. Independent surveyors also described women as sounding less competent than men when discussing research [22].

As a result of how they are perceived, women often change their behaviour around male colleagues. In settings with a male majority or dominance, women are less likely to share their opinions towards group decisions made by consensus [23]. Because men are more likely to fill senior positions at academic institutions, the gender perception gap is limiting the progress of women in science, and thus needs to be addressed. One solution is to put women in positions with increased visibility, where they have a greater influence on junior female scientists, helping to eliminate the misconception that women are less competent scientists than men.

### Measures of Visibility

There are many venues through which a scientist can increase their visibility and subsequently their level of professional recognition, including publishing research in a peer-reviewed journal [4][6][9][24], receiving awards [25][26][27], lecturing at universities or other academic institutions [1][3][17], and speaking or organizing symposia at conferences [13][18][28][29]. In all of these cases, however, women have been statistically underrepresented.

Since many papers have focused on publications, awards, and employment, we wish to more deeply examine the representation of female scientists in symposia at professional society conferences. We chose to focus on symposia only, rather than all general and plenary talks, because organizers specifically invite symposium speakers. Therefore, there is a greater chance that gender bias could be introduced into the speaker list. Simultaneously, symposia showcase speakers in a more prestigious venue and thus present an opportunity to reject negative gender schema.

Quantifying the ratio of female-to-male conference speakers can also be considered a measure of novel collaboration, since inviting scientists to speak leads to academic relationships being built between organizers and speakers, if such relationships were not already present. The personal aspect of conference invitations could coincide with the findings that international collaboration might “level the playing field” for women [8].

Finally, this is a reasonable venue to assess gender parity, since gender bias has previously been identified in how others perceive conference abstracts. For example, the conference abstracts of male speakers were perceived by conference participants to be of higher scientific quality than those of women [30].

### Assessing Gender Bias at the Symposium, Conference, and Society Level

Presenting in symposia at scientific conferences provides speakers with networking prospects, peer recognition, and future professional endeavour opportunities [28]. Assessing the influence of women at conferences requires investigation at different levels of organization within scientific societies. At a fine-scale level, symposium speakers are invited at the discretion of the organizers, based on whom the organizers recognize as having made great strides in the subject of interest. Thus, the ratio of female-to-male speakers is a basal assessment of gender equality.

At a conference-wide level, comparing the ratio of female speakers to male speakers when the organizers were all male is a fundamental way to assess the message that was being portrayed to junior scientists about the importance of women in science [28]. Ultimately, the gender ratio of the organizing committee can influence the gender ratio of speakers [29].

At the professional society level, the invitation process represents an opportunity for societies to highlight and promote diversity within their ranks. Thus, quantifying the visibility of women speakers at conferences in relation to the number of women organizers is a comprehensible method of assessing the representation of women in science more generally [13]. This metric is accessible through society websites, where lists of speakers and organizers from past conferences have been documented.

We examined the relationship between the gender ratio of symposium organizers and of speakers within symposia. As we are marine conservation biologists, we examined these dynamics within our ‘home’ societies. We focused on data from the Society for Conservation Biology and the American Society of Ichthyologists and Herpetologists. We hypothesized that there would be a positive correlation between the number of female organizers and subsequent female speakers, reflecting either gender assortment among professional networks or proactive motivation to achieve better gender representation within symposia. Secondly, due to increased awareness for gender equality in science and increased involvement of women in the STEM field, we predicted that there would be more female organizers and presenters at both conferences over time.

## Methods

We collected information on symposia organizers and speakers from conferences held by the Society of Conservation Biology (SCB) Global chapter and from the American Society of Ichthyologists and Herpetologists (ASIH) annual meetings. The names of organizers and presenters were collected from scientific programs that are accessible online through the societies’ websites (https://conbio.org/conferences/about-scb-meetings and http://www.asih.org/meetings, respectively). The two conferences were considered separately in order to retain the resolution of the data between groups. By analyzing the conferences individually, we can show that, even though they may share membership and are temporally correlated, the organizing committee itself has the strongest influence over the gender ratio. We can thus quantify the variation between conferences to avoid making generalizations, which is less condemning to the scientific community as a whole.

Some programs were not available online, however, and have not been retained in university libraries or by the conference administration. Further, some scientific programs were not available due to link rot. Therefore, our data consists of organizers and speakers from SCB Global conferences from 1999, 2002, 2006, 2007, 2009, 2010, 2011, 2013, 2014 (Society for Conservation Biology Oceania chapter, included because of an all-female lead and impressive strides in diversity equity), and 2015. Data from ASIH included 2005, 2006, 2007, 2008, 2009, 2010, 2011, 2013, 2014, and 2015. Note that after 2011, the SCB Global conferences became biannual, and that the 2012 ASIH conference was held in conjunction with the World Congress of Herpetology, but no program has been retained by the society. In total, 289 symposia were assessed from SCB Global conferences with 612 organizers and 1,958 speakers; 56 symposia, 132 organizers, and 933 speakers were assessed from ASIH conferences. The membership of SCB is approximately 5,000 individuals, and 1,500 for ASIH. While SCB does not publish information on the diversity of their membership, ASIH published a quantification of the gender and ethnic diversity of their society in 2015. At that time, 68.69% of members were male, 30.14% were female, and 1.17% did not disclose their gender.

To infer gender, speakers and organizers were researched online. Since our data set was a manageable size, we could investigate individuals manually. Most scientists have university, ResearchGate, Google+, or LinkedIn profiles with their photograph that indicate their gender. Any gender error bias would have been random (i.e. “John” would be presumed male when actually female as often as “Sally” would be presumed female when male): there is no expectation that cross-gender mistakes would introduce a bias into our analyses. As a first approximation, we assumed gender to be binary (woman or man), despite our recognition that gender is a cultural construct and better viewed along a gradient influenced by personal perspectives [31]. The binary approach was more conducive to analysis, since the gradient of gender is so diverse, so we were unable to consider additional identities due to analytical limitations. Thus, we explicitly included transgender individuals as the gender they chose to identify as.

To reduce bias in our study, we ensured there was not a significant overlap in attendance between the two conferences by presenters or organizers. In other words, we confirmed that there was a low percentage of female organizers or speakers who participated at both conferences in the same year, as this would have influenced our ability to use conference participation as an accurate measure of visibility. We cross-referenced the names of organizers and speakers in the scientific programs for the conferences of both societies.

We did not differentiate between the academic positions of the speakers (i.e., professors, postdoctoral researchers, and students at the PhD, Masters, or Bachelor level). This parameter is supported in similar studies, which suggested no significant difference in the gender ratio of presenters among students and higher-level academics at an annual meeting of an ecological society [13]. We also did not consider the nationality or institution of the speakers and organizers, as these variables do not influence the competency scores of women during the peer-review process [19].

Lastly, we did not exclude speakers who were also organizers, as we aimed to consider the general stage presence of women, regardless of their higher roles. While women organizers were likely to also be speakers in their symposium, we considered females who both organized and presented to still have an overall positive effect towards reducing gender bias [28].

We performed multivariate regression analyses using the statistical program R [32], considering a p-value with a confidence interval greater than 95% significant (p<0.05). We assessed the relationship among the number of female organizers, speakers, and the total number of organizers and speakers at both the SCB Global and ASIH conferences. The “total number of organizers/presenters” refers to the cumulative number of both women and men involved in one year or symposium. We also examined whether the gender ratios of organizers and speakers have changed over time. The data was jittered for better visualization.

Finally, we estimated the number of female speakers that each additional female organizer after the first would bring to the table by calculating the slope associated with the number of speakers as the number of organizers increased.

## Results

There was not a significant overlap of participants who were organizers and/or presenters at both ASIH and SCB Global conferences in the same year. While there was some overlap in attendance, less than 1% of organizers or presenters from SCB actually organized symposia or spoke in a symposium at ASIH, and vice versa.

At SCB Global conferences, 36.4% of organizers and 31.7% of presenters for the entire conference (all symposia cumulatively) were female. At the ASIH conferences, 19.1% of organizers and 28% of presenters for the entire conference (all symposia cumulatively) were female (Table 1). Since ASIH published membership information, we compared our results to the overall female membership. The percentage of female presenters and organizers is less than the percentage of the membership that is female (30%), suggesting there is a skew towards increased male participation.

**Table 1:** Total number of organizers and presenters at both conferences per year, and the percentage of organizers and presenters per conference that were female. These numbers are based on symposia cumulatively across the yearly conferences.

Conference	Year	Total Organizers	% Female Organizers	Total Presenters	% Female Presenters
SCB	1999	12	25	41	26.8
SCB	2002	43	39.5	152	26.3
SCB	2006	45	28.9	157	22.9
SCB	2007	48	35.4	169	27.2
SCB	2009	49	20.4	76	23.7
SCB	2010	81	30.9	247	26.7
SCB	2011	79	31.6	242	28.1
SCB	2013	103	45.6	315	38.7
SCB	2014	40	70	152	56.6
SCB	2015	141	36.2	407	39.8
**SCB**	**Total**	**641**	**36.4**	**1958**	**31.7**
ASIH	2005	12	8.3	93	24.7
ASIH	2006	13	30.8	100	30
ASIH	2007	8	0	51	13.7
ASIH	2008	9	22.2	69	20.3
ASIH	2009	30	16.7	175	24.6
ASIH	2010	17	41.2	155	44.5
ASIH	2011	13	15.4	80	30
ASIH	2013	11	27.3	92	32.6
ASIH	2014	5	0	39	23.1
ASIH	2015	14	28.6	79	36.7
**ASIH**	**Total**	**132**	**19.1**	**933**	**28**

The number of female organizers per conference, female presenters per conference, total number organizers per conference, and total number of presenters per conference for both conferences were not normally distributed. As such, the conferences were considered independent, and Mann-Whitney U tests for non-parametric and non-normal data were conducted to compare the percentage of female organizers and female presenters between both conferences. There was a significant difference between the total numbers of organizers per conference (p<0.01), the total numbers of presenters per conference (p = 0.04), and the percentages of female organizers per conference (p = 0.01), but there was not a significant difference between the percentages of female presenters per conference (p = 0.57) (Table 1).

Based on the slope of the number of female speakers per symposium for every additional female organizer per symposium, we observed that every additional female organizer after the first increased the number of female presenters at each symposium by 95% (SCB) and 70% (ASIH). Further, the number of female presenters per symposium increased continuously as the number of female organizers per symposium did.

The total number of organizers per conference increased significantly over time at the SCB conferences (p<0.01, multiple R^2^ = 0.56), as did the total number of presenters per conference (p<0.02, multiple R^2^ = 0.52). However, the percentage of female organizers per conference and the percentage of female presenters per conference did not increase significantly over time (p = 0.17, multiple R^2^ = 0.22 and p = 0.0504, multiple R^2^ = 0.404, respectively).

At ASIH conferences, the total number of organizers per conference (p = 0.77, multiple R^2^ = 0.011), the total number of presenters per conference (p = 0.61, multiple R^2^ = 0.033), the percentage of female organizers per conference (p = 0.73, multiple R^2^ = 0.016), and the percentage of female presenters per conference (p = 0.25, multiple R^2^ = 0.167) did not increase significantly over time.

### Relationships between Organizer Gender Ratio and Speaker Gender Ratio per Symposium

**Fig 1:**
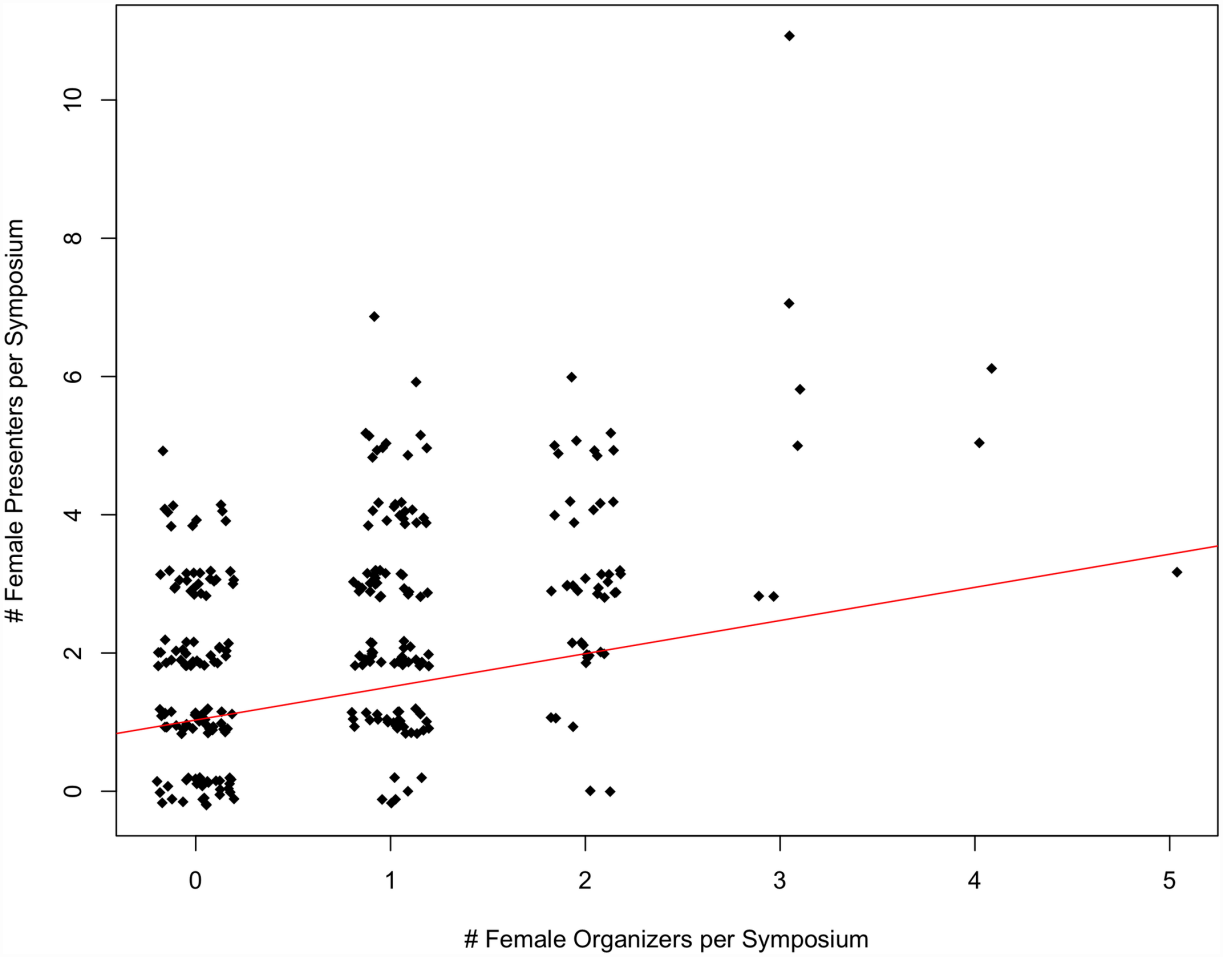
The number of female presenters per symposium as a function of the number of female organizers per symposium at SCB Global conferences between 1999–2015 (p<0.01, multiple R^2^ = 0.22). Note that outliers were removed and re-added to assure they did not influence the significance of the regression relationship.

**Fig 2:**
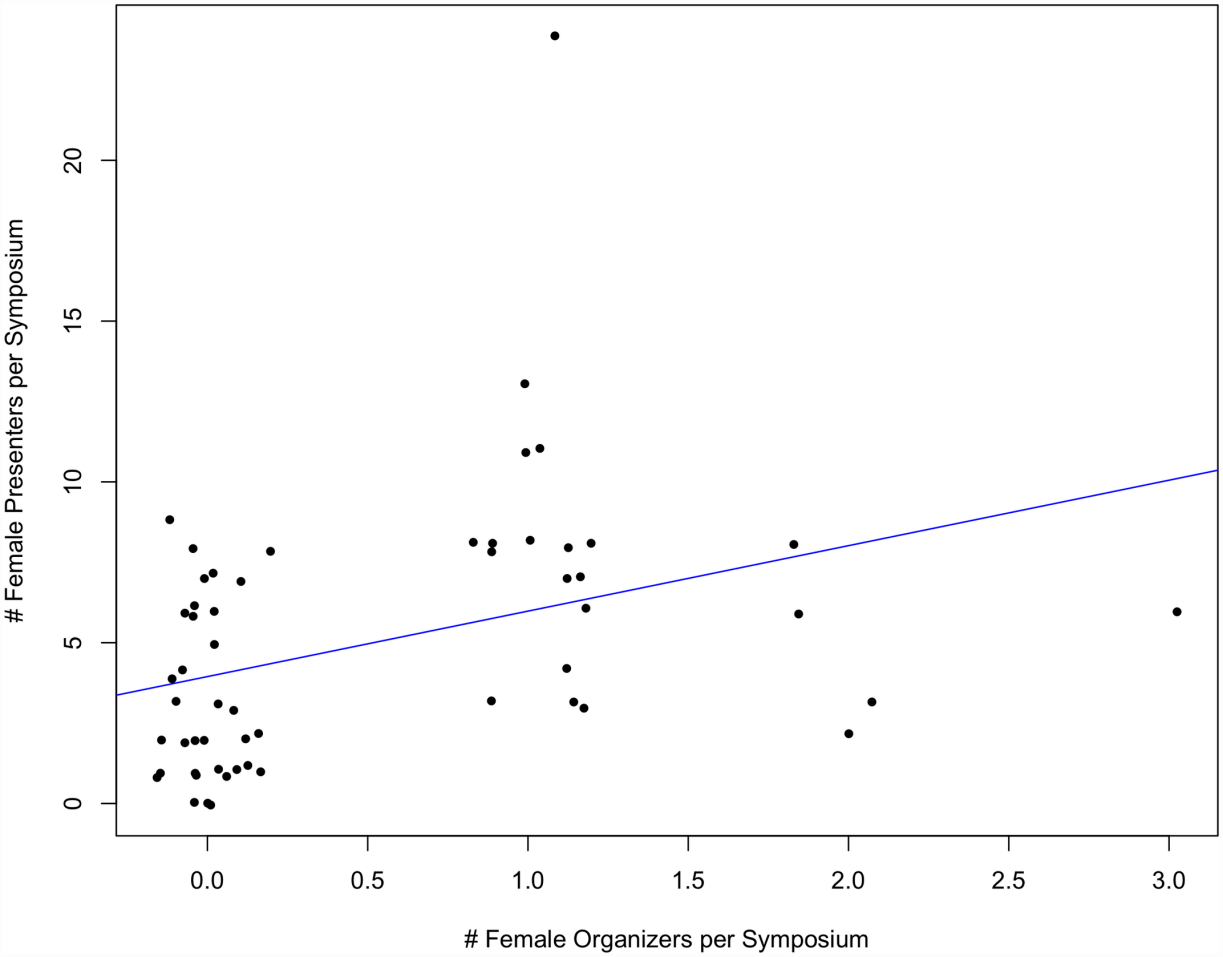
The number of female presenters per symposium as a function of the number of female organizers per symposium at ASIH conferences between 2005–2015 (p<0.01, multiple R^2^ = 0.12). Note that outliers were removed and re-added to assure they did not influence the significance of the regression relationship.

At both SCB and ASIH conferences between 1999 and 2015, there was a strong, significant, positive correlation between the number of female organizers per symposium and the number of female presenters per symposium (p<0.001, multiple R^2^ = 0.22 and p<0.001, multiple R^2^ = 0.12, respectively) (Figs 1 and 2).

**Fig 3:**
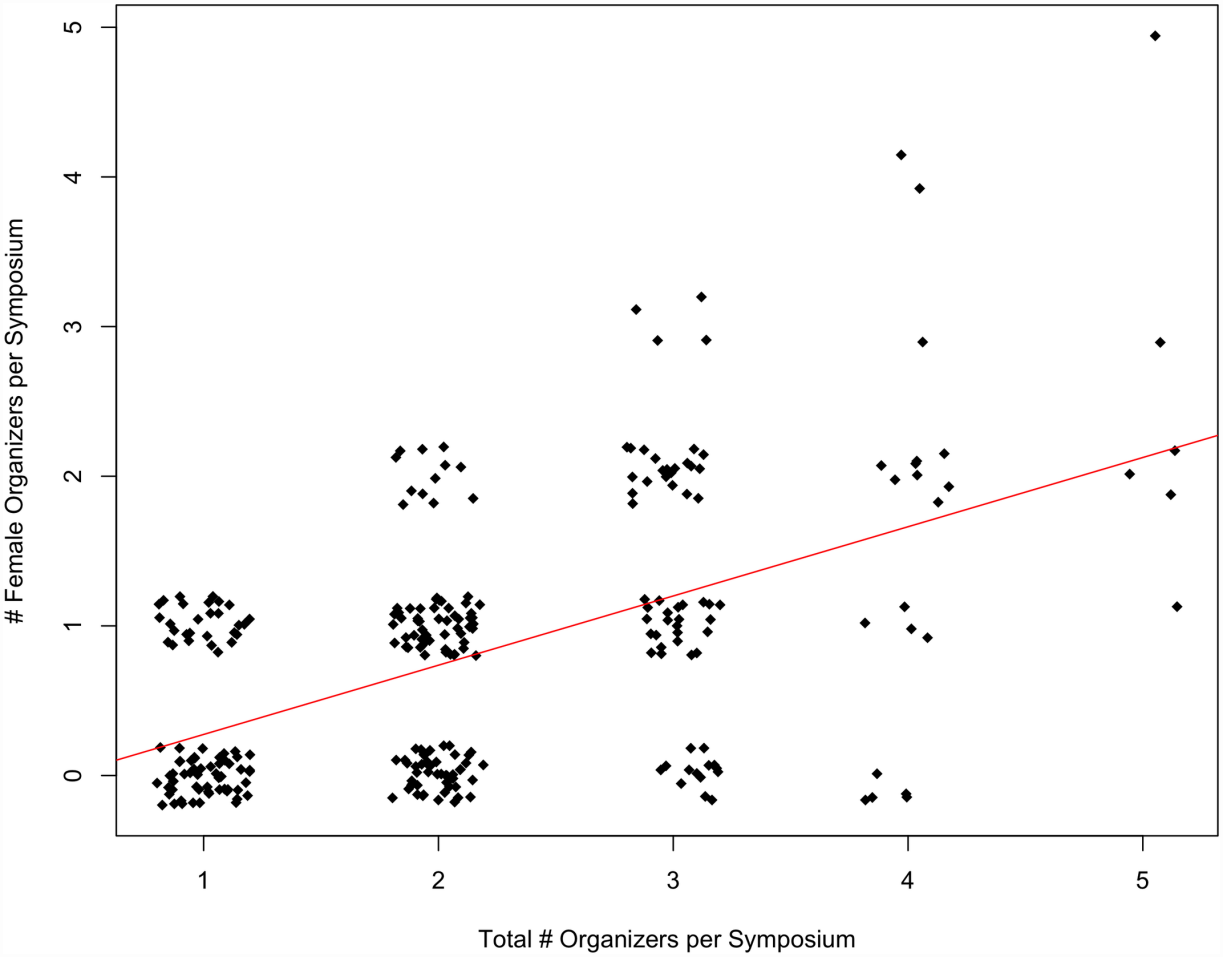
The number of female organizers per symposium compared to the total number of organizers per symposium at SCB Global conferences from 1999–2015 (p = 0.07, multiple R^2^ = 0.28).

**Fig 4:**
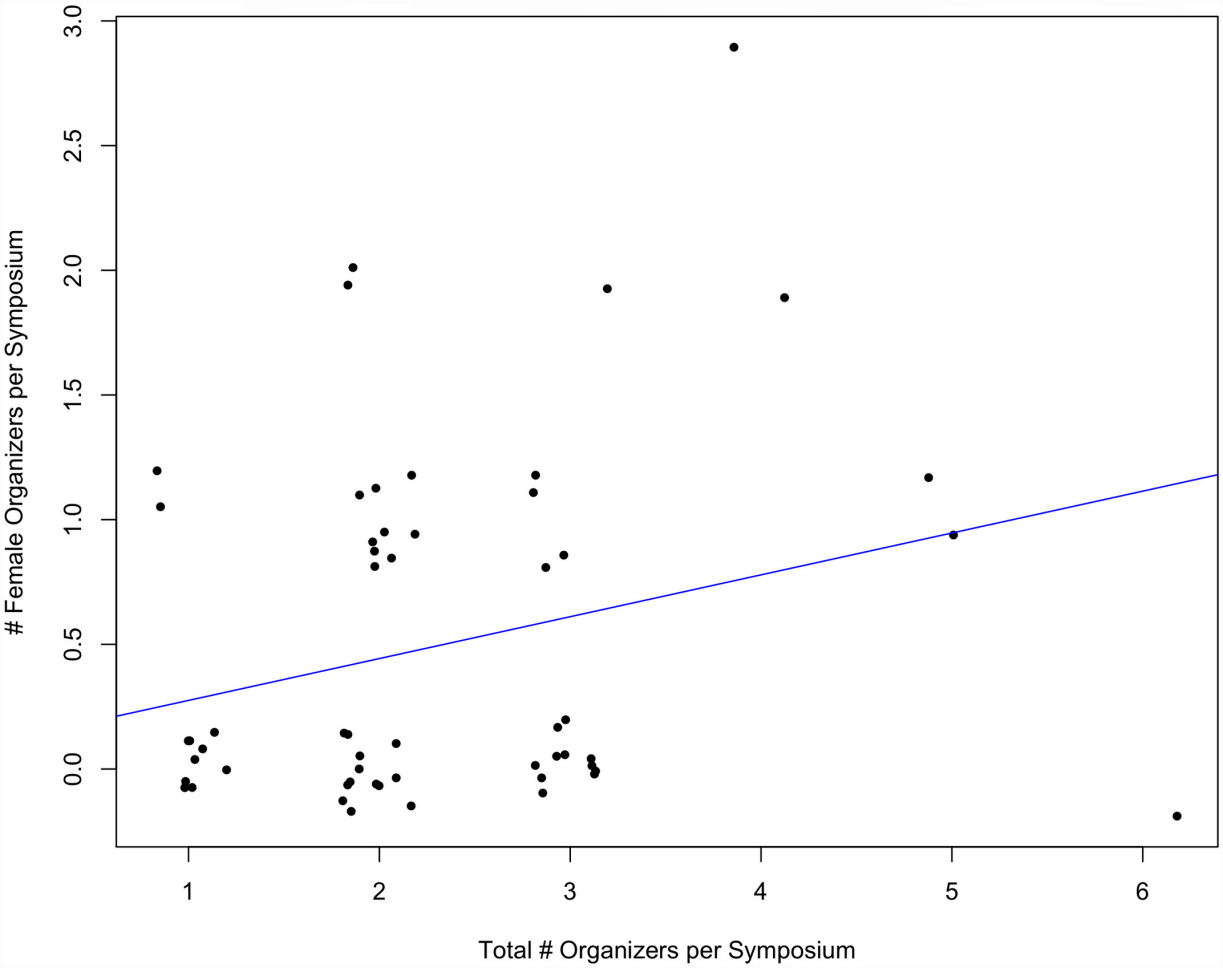
The number of female organizers per symposium compared to the total number of organizers per symposium at ASIH conferences from 2005–2015 (p = 0.47, multiple R^2^ = 0.063).

There was also a significant, positive correlation between the number of female organizers per symposium and the total number of organizers per symposium at the SCB Global conferences (p = 0.07, multiple R^2^ = 0.28) (Fig 3). In other words, as the organizer pool increased in size, the opportunities for female organizers increases significantly. Yet, this was not the case for ASIH conferences. As the number of organizers per symposium increased, the number of female organizers per symposium did not increase significantly (p = 0.47, multiple R^2^ = 0.063) (Fig 4).

**Fig 5:**
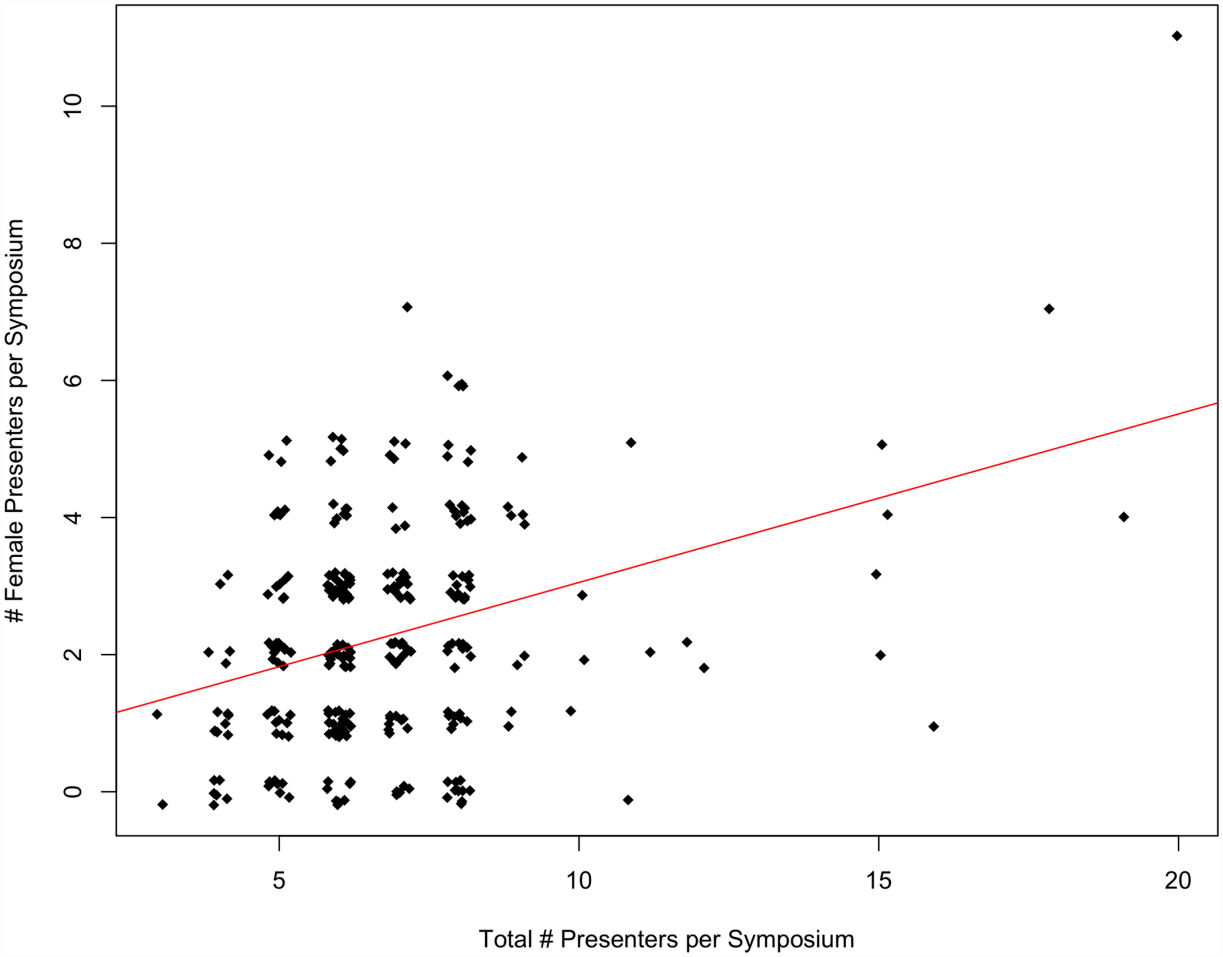
The number of presenters per symposium compared to the total number of presenters per symposium at SCB Global conferences from 1999–2015 (p = 0.04, multiple R^2^ = 0.12).

**Fig 6:**
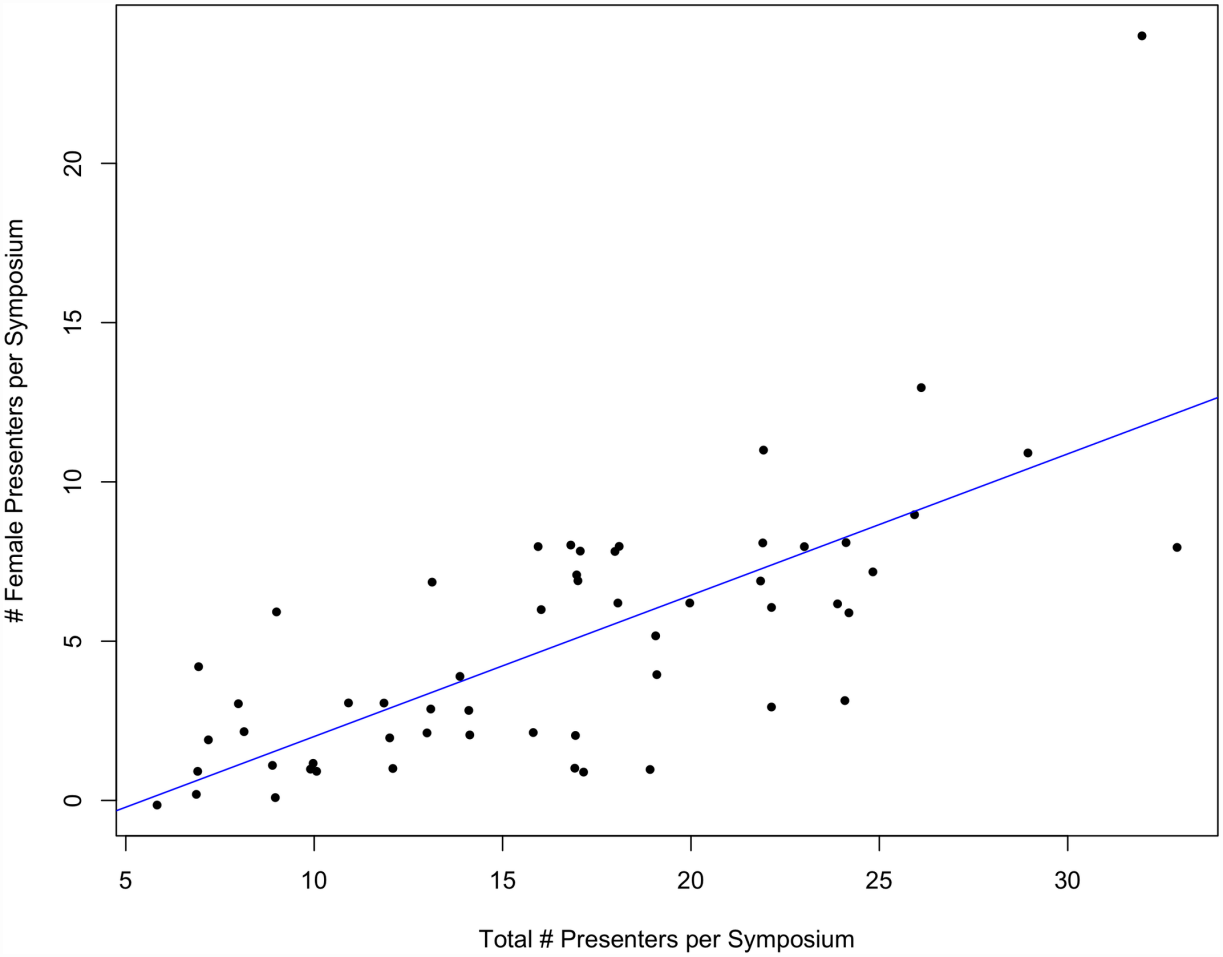
The number of female presenters per symposium compared to the total number of presenters per symposium at the ASIH conferences from 2005–2015 (p = 0.02, multiple R^2^ = 0.51).

At both conferences, there was a significant, positive correlation between the number of female presenters per symposium and the total number of presenters per symposium (SCB p = 0.04, multiple R^2^ = 0.12; ASIH p = 0.02, multiple R^2^ = 0.51) (Figs 5 and 6). So, as the size of the pool of presenters increased, there were significantly more women presenting.

### Temporal Trends

**Fig 7:**
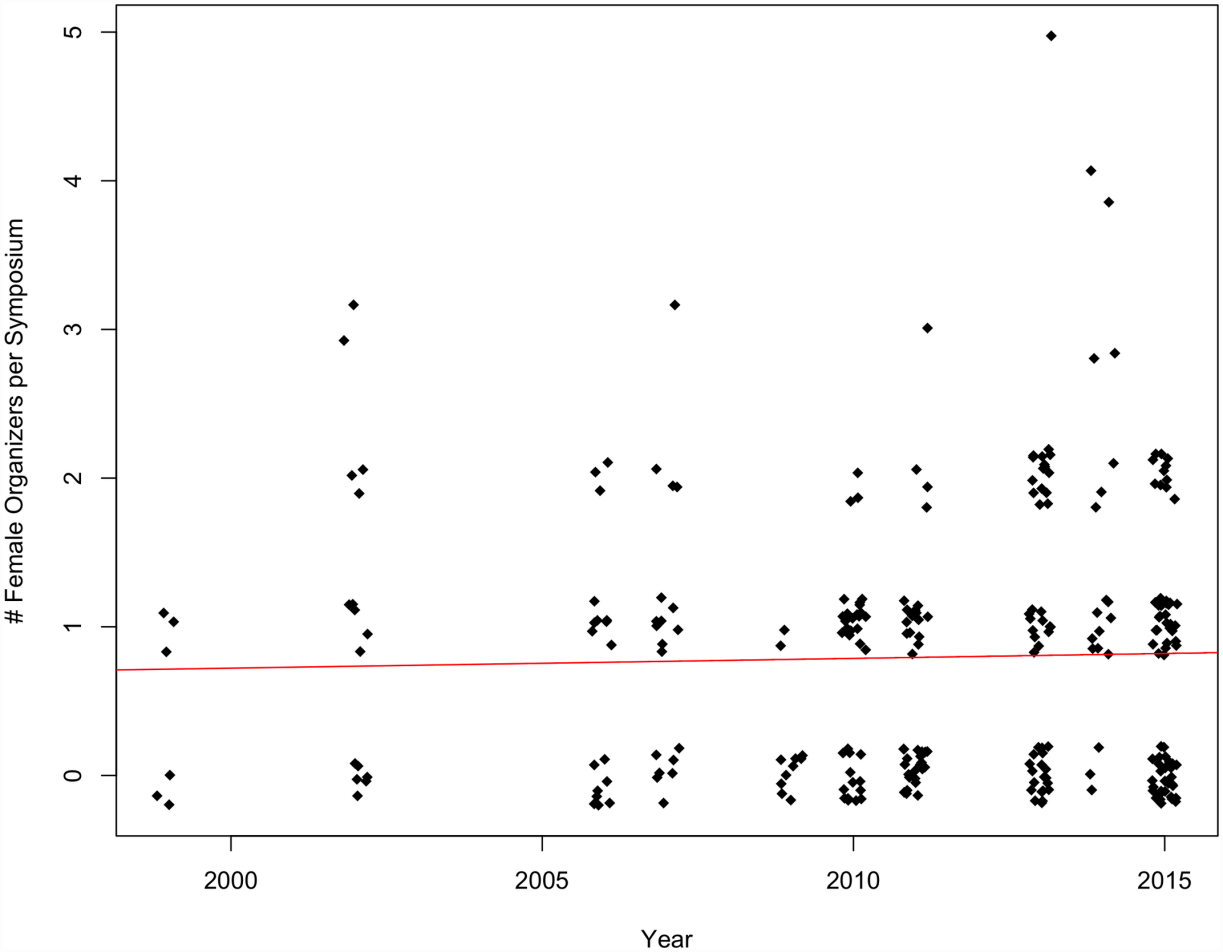
The number of female organizers per symposium over time at SCB Global conferences from 1999–2015 (p = 0.63, multiple R^2^ = 0.0009).

**Fig 8:**
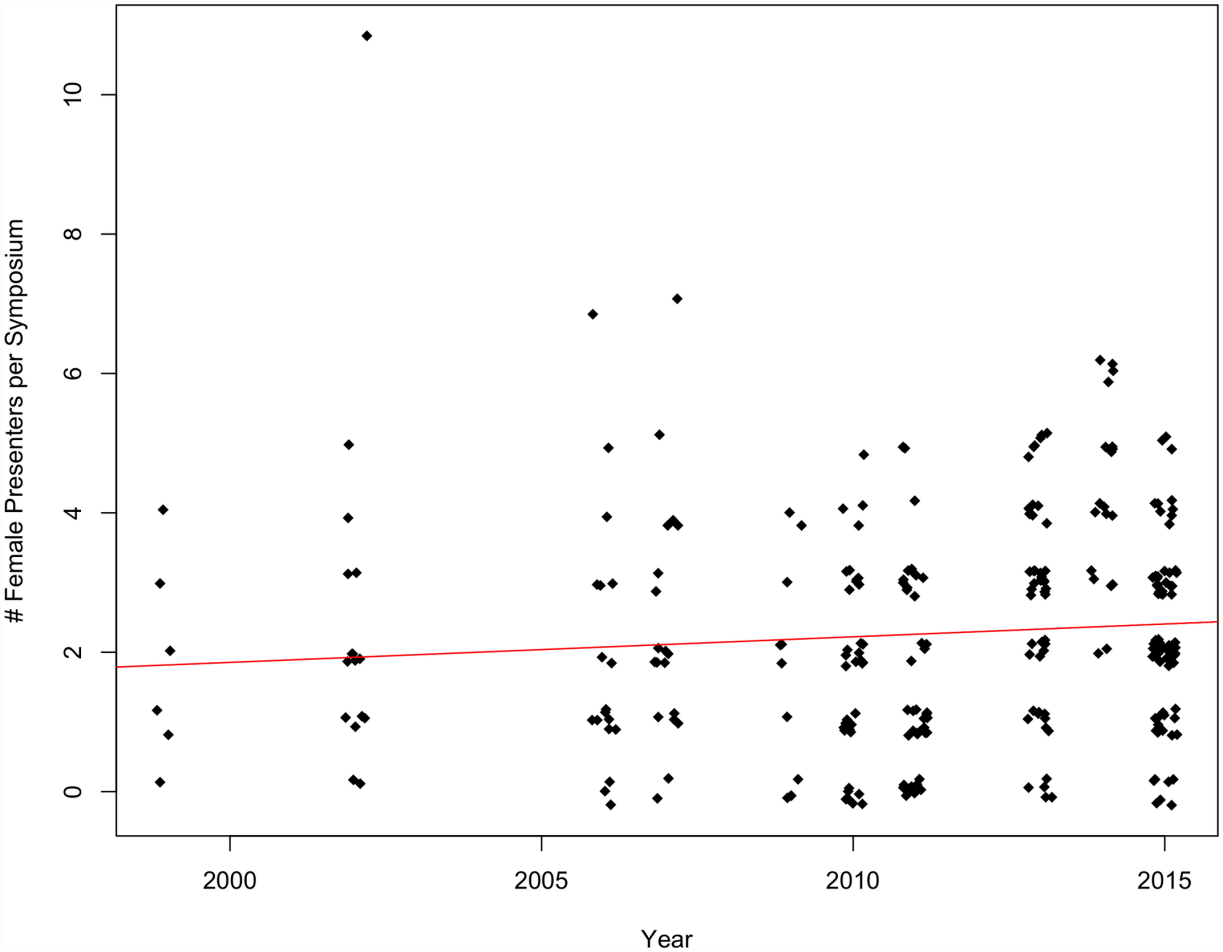
The number of female presenters per symposium over time at SCB Global conferences from 1999–2015 (p = 0.14, multiple R^2^ = 0.008).

**Fig 9:**
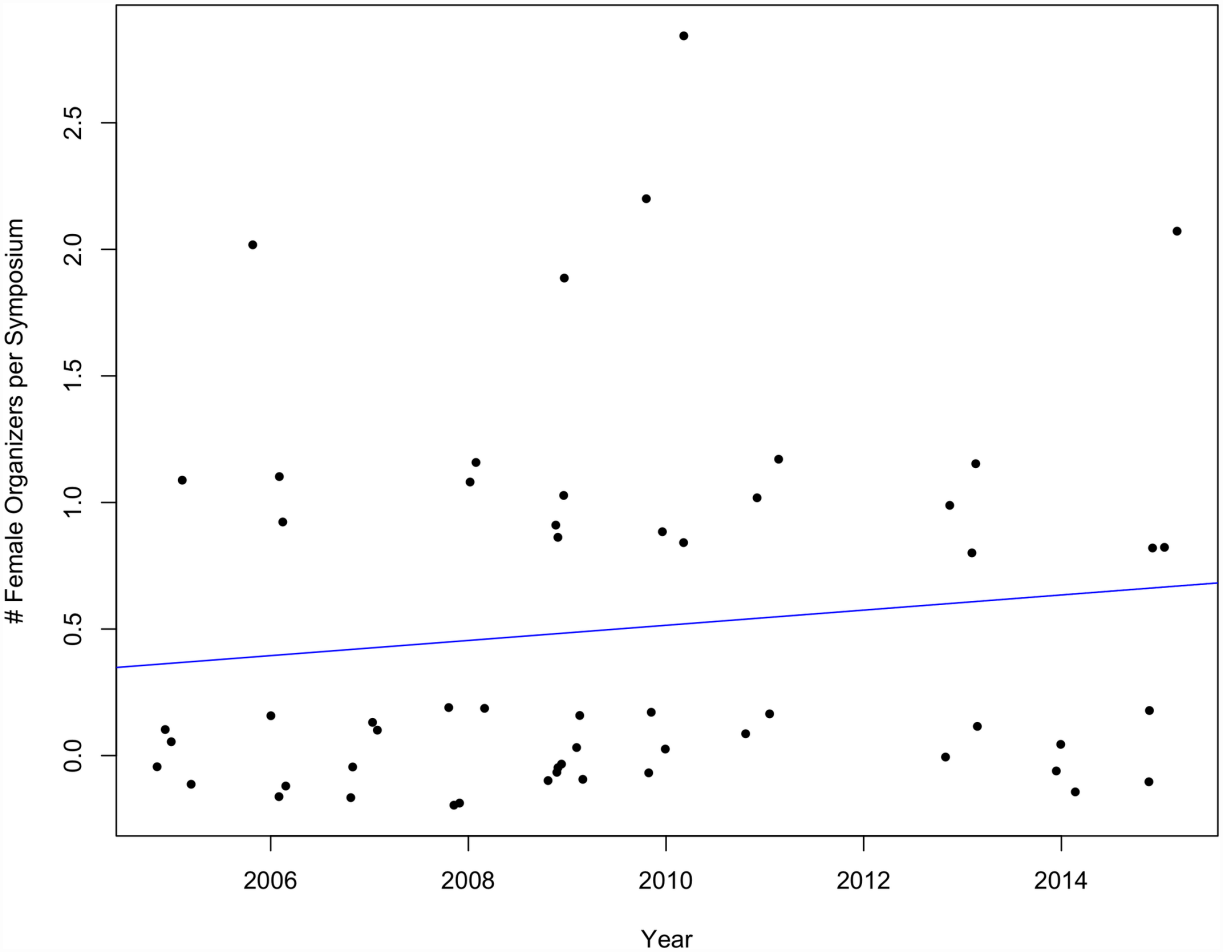
The number of female organizers per symposium over time at ASIH conferences from 2005–2015 (p = 0.36, multiple R^2^ = 0.016).

**Fig 10:**
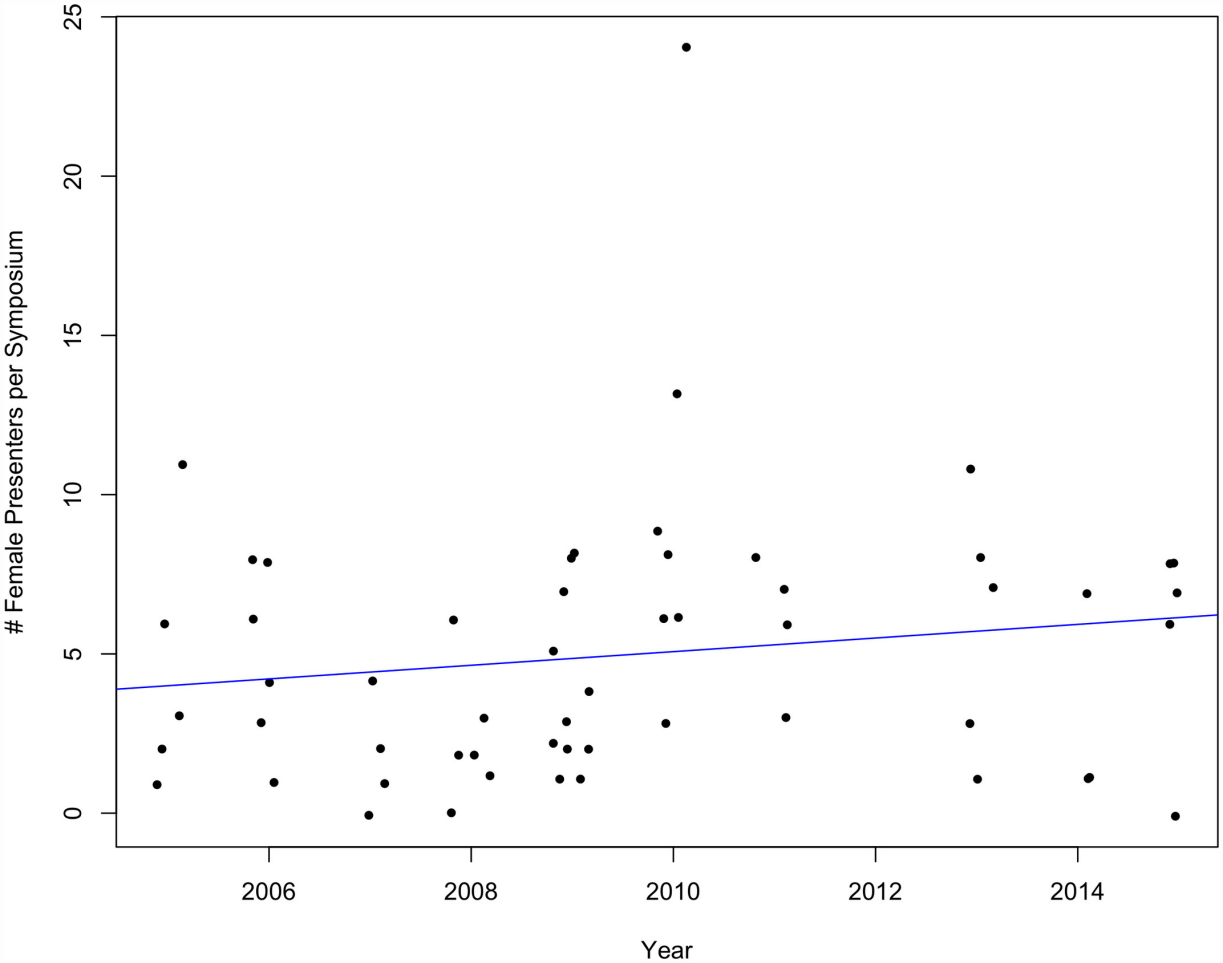
The number of female presenters per symposium over time at ASIH conferences from 2005–2015 (p = 0.26, multiple R^2^ = 0.025).

Neither the number of female organizers per symposium nor female presenters per symposium increased significantly over time at SCB Global conferences between 1999 and 2015 (organizers p = 0.63, multiple R^2^ = 0.0009; presenters p = 0.14, multiple R^2^ = 0.008) (Figs 7 and 8) nor ASIH conferences between 2005 and 2015 (organizers p = 0.36, multiple R^2^ = 0.016; presenters p = 0.26, multiple R^2^ = 0.025) (Figs 9 and 10).

Additionally, the total number of presenters per symposium and the total number of organizers per symposium at SCB Global conferences have decreased significantly over time (p<0.01, multiple R^2^ = 0.17 and p<0.01, multiple R^2^ = 0.029, respectively), suggesting that while the cumulative total numbers of presenters and organizers at the SCB Global conferences have increased over time (Table 1), it has been through more numerous but smaller symposia. This was not the case for ASIH conferences, where the total numbers of presenters and organizers per symposium did not change significantly over time (p = 0.8, multiple R^2^ = 0.0015 and p = 0.36, multiple R^2^ = 0.016, respectively).

## Discussion

### More Female Organizers Promote More Female Speakers

Our results show a strong relationship between women’s participation in the organization of symposia and opportunities for women to participate in symposia. This relationship is present at both professional societies. As these societies have several thousands of members worldwide, they represent a tremendous opportunity for career advancement for scientists. When women conference organizers provide a platform for other women to highlight their work, the organizers do not “pull up the ladder” or reduce opportunities for other women, helping to staunch some of the leaks in the pipeline. We have shown that increasing the number of female organizers leads to a continuous increase in the number of female presenters. Each additional female organizer per symposium has a nearly one-to-one increase in female speakers (SCB: 95% speakers per organizer per symposium; ASIH: 70% speakers per organizer per symposium).

Our results support emerging research that identifies gender-based disparities within the sciences that are based on the orientation of individuals towards others, rather than any deviation in ability or achievement [33]. This may indicate that women are more aware of avoiding gender bias, that some males lack recognition for female scientists, or that gender disparity cannot be disregarded in academia [9][34]. The positive, significant relationships observed at both SCB Global and ASIH conferences between the numbers of female organizers and female presenters per symposium indicates that female organizers are more likely to oversee symposia with female speakers. At both conferences, as the total number of presenters and organizers per symposium increased, so did the number of female presenters and organizers. Yet, the female-to-male ratio did not increase significantly, suggesting that there is a defect of female speakers and organizers.

### Larger Symposia Provide Greater Opportunity for Diversity

Symposia with larger pools of speakers and organizers are significantly more likely to include women in both categories, suggesting inclusivity may be stimulated by larger symposia with greater topic generality. Increasing the total number of organizers per symposium had a significant positive effect on the number of female organizers at SCB conferences, and increasing the total number of presenters per symposium had a significant positive effect on the number of female presenters at both conferences.

The SCB Global conferences have continuously increased in size over the past two decades, resulting in the administrative decisions to make the general, or Global, assembly biannual and to initiate region-specific sub-conferences. Despite overall increases in the number of organizers and speakers per conference over time, the numbers of organizers and speakers per symposia decreased significantly over time. Smaller, potentially more specialized, symposia are significantly less likely to include women in their programming, introducing an air of exclusivity to the platform. Simply put, larger symposia might help to balance gender participation at conferences.

On the other hand, ASIH conferences remain annual events and have not had a significant increase over time in the total number of organizers or speakers per conference. Further, while there was a significant increase in female presenters as the total number of presenters per symposium increased, there was no statistical benefit to women when the organizational team was expanded. As such, reinstating larger, more encompassing symposia to allow greater opportunity for women to participate—akin to the recommendation above for SCB—would not necessarily be applicable or successful for ASIH. This is an ideal example of how diverse the issue of gender disparity is across different segments of academia, and that issues with the overarching culture must be addressed in order for uniform, widespread change.

### Continuing Temporal Inequity

There remains work to be done in achieving gender equality, as the number of female organizers and female presenters per symposium has not increased significantly over time at either conference. This gender difference is not only a factor of unbalanced invitation, but also relates to who accepts them. While women may be more likely to consider the gender of the speakers and seek women presenters, men might be less likely to accept an invitation from a woman [28]. Women have also been observed to have a higher rate of rejecting invitations to speak in symposia, furthering any divergence in the gender ratio [18]. As such, we should also consider other barriers to participation, and in so doing, identify a suite of solutions to help address representation biases.

#### Increased Professional Interaction between Men and Women

Creating positive environments were men and women can interact professionally, both at conferences and at home institutions, might help increase the number of women organizers and speakers over time via more diverse professional networks. “Friendship bonuses” occur when applicants for peer review that are acquainted to the reviewer are given more opportunities [35] and are considered more competent scientists [19]. In the context of a conference, these perks could manifest themselves in the form of male organizers being more likely to invite male associates to present in their symposia, especially if they have been restricted from interacting with women professionally due to stereotype barriers at their home institution. This can be addressed by conferences by including mandatory quotas of invited female scientists, which will increase the professional interactions between men and women and potentially have positive, long-term effects.

### The Glass Ceiling

Stereotypes limit the success of women in patriarchal fields; women generally face underrepresentation in those subjects [13]. While the performance gap between men and women in STEM disciplines has been decreasing over the past two decades, the professional goals men and women set for themselves based on their self-conception remain disparate [15]. This “glass ceiling” effect, where progress is limited by inequality in salary and opportunities, contributes to unequal ratios of women-to-men that receive recognition in academic programs (i.e. citations, awards, or the perceived value of their research) [13][36]. This perception or recognition can be quantified and acknowledged by being invited to present at a symposium.

The discrepancy between the success of men and women in science is often attributed to the different lifestyle choices men and women make—primarily the decision to have children or not [4]. However, this is a nuanced issue; in general there is compelling evidence to suggest equal levels of productivity between mothers and non-mothers, as they publish equal amounts of peer-reviewed literature [35][37][38].

### Barriers to Participation

In addition to (and potentially as a consequence of) stereotyping, there are numerous barriers preventing women from participating in scientific conferences. The following barriers accentuate the so-called “pool problem”, where the number of female applicants is disproportionately lower than the number of females succeeding (for example, by receiving doctoral degrees [17]). The performance of women is impaired by a predominant mentality of inferiority in academia, increasing male motivation to avoid inviting female speakers [3][39].

To better translate our findings into a suite of real world actions, we present the following suggestions to scientific societies for improving gender equality. They are focused towards increasing the number of invitations women receive and accept to speak at conferences. Both the Society for Conservation Biology and the American Society of Ichthyologists and Herpetologist have taken steps to improve gender representation at their conferences, but revitalized efforts are required to avoid prolonged inequality.

#### Facilitating Travel

One of the major hurdles of conference participation is cost. Women professors are typically paid less than their male counterparts [17][40] and, particularly salient in the context of increasing participation of junior members, receive smaller start-up packages [41]. To help alleviate the disproportionately greater financial burden women face when attending meetings, societies should allocate special funds to help subsidize women participants. For example, The Society for Comparative Biology has established the Dorothy Skinner Fund, which provides conference travel funding for women graduate students and postdoctoral researchers [42].

#### Child-Friendly Conferences

Mothers in the U.S. are more likely to leave science than single women, whereas parenthood does not have a significant effect on the retention of men in STEM fields [17]. As women are disproportionately responsible for childcare, this manifests itself at conferences as a supplementary set of obligations to manage in addition to attending talks, networking, and participating in workshops. Conference organizers could help junior members of both genders by providing subsidized childcare (which SCB Global has offered at meetings) and help new mothers by designating dedicated nursing rooms. Similarly, conference events and plenary events housed during the day provide opportunities for parents to attend and for conferences to be child-friendly. No scientist at any level should be faced with a “needless clashing” of their career path and their familial aspirations [43].

#### Promoting Women to Organize More Symposia

The most important finding from our results was that there is a strong, positive relationship between the number of women involved in organizing symposia and the number of women speaking in those symposia. Conference organizers should therefore encourage and actively recruit talented female scientists to submit symposium proposals for those conferences. For example, the 2014 SCB Oceania meeting in Fiji had the highest percentage of women speakers per symposium (56%) and organizers per symposium (72%) of all the meetings we examined. This was likely the positive influence of women chairs and head committee members. This conference can be considered an example of the downstream implications of diverse, considerate leadership. The conference committee for this event actively sought out women organizers for their symposia, thereby realizing an effective and energetic meeting environment.

#### Codes of Conduct

A rigid, enforced, and mandatory Code of Conduct should be established for all scientific conferences in order to maintain the comfort and safety of all participants. Codes of Conduct should solidify what constitutes unacceptable behaviour, including zero-tolerance for abuse towards minorities, women, and differently abled participants at conferences. This includes verbal derogatory or sexualizing comments, as these are not isolated incidents. For example the chairs of SCB’s International Marine Conservation Congress 2016 have included a thorough Code of Conduct in the registration process, where delegates and presenters alike must confirm their understanding and adherence to the Code of Conduct before acquiring approval of their attendance [44]. For a Code of Conduct to be effective, the enforcement of regulations and the protocol for how to report an incident must be clear. With this, an organization signals to its members that it has consciously committed to protecting them during the proceedings of their conference.

## Conclusion

For true progress in the scientific community towards gender equality, women must be recognized as scientists in academic settings rather than just as women [10]. With hopes of highlighting the successes of women in science and promoting equal opportunity for scientists regardless of gender, our research aids in identifying gaps in our community that can be ameliorated through collaboration and tolerance. Lessening the impacts of the leaky pipeline effect in the biological sciences will be a matter of increasing social belonging and exposure of women at conferences, subsequently “promoting opportunities for peer networking” [15]. Increasing exposure of women is our hypothesized method for increasing retention. We have also solidified the methodology for future studies to examine the gender ratio at conferences within other STEM disciplines. By conducting similar research along other axes of diversity (i.e. race, sexual orientation, geographic representation, ability status, and the intersection of these axes), we hope to provide a framework for improving the representation of diversity within the broader scientific community.
